# Cognitive reserve and cognitive performance of patients with focal frontal lesions

**DOI:** 10.1016/j.neuropsychologia.2016.12.028

**Published:** 2017-02

**Authors:** Sarah E. MacPherson, Colm Healy, Michael Allerhand, Barbara Spanò, Carina Tudor-Sfetea, Mark White, Daniela Smirni, Tim Shallice, Edgar Chan, Marco Bozzali, Lisa Cipolotti

**Affiliations:** a Centre for Cognitive Ageing and Cognitive Epidemiology, University of Edinburgh, Edinburgh, UK; b Department of Psychology, University of Edinburgh, Edinburgh, UK; c Department of Neuropsychology, National Hospital for Neurology and Neurosurgery, London, UK; d Neuroimaging Laboratory, Santa Lucia Foundation, Rome, Italy; e Department of Neuroradiology, National Hospital for Neurology and Neurosurgery, London, UK; f Dipartimento di Scienze Psicologiche, Pedagogiche e della Formazione, Università degli Studi di Palermo, Palermo, Italy; g Institute of Cognitive Neuroscience, University College London, UK; h International School for Advanced Studies (SISSA-ISAS), Trieste, Italy

**Keywords:** Cognitive reserve, Frontal lesions, Education, Literacy attainment, Cognitive performance, Age

## Abstract

The Cognitive reserve (CR) hypothesis was put forward to account for the variability in cognitive performance of patients with similar degrees of brain pathology. Compensatory neural activity within the frontal lobes has often been associated with CR. For the first time we investigated the independent effects of two CR proxies, education and NART IQ, on measures of executive function, fluid intelligence, speed of information processing, verbal short term memory (vSTM), naming, and perception in a sample of 86 patients with focal, unilateral frontal lesions and 142 healthy controls. We fitted multiple linear regression models for each of the cognitive measures and found that only NART IQ predicted executive and naming performance. Neither education nor NART IQ predicted performance on fluid intelligence, processing speed, vSTM or perceptual abilities. Education and NART IQ did not modify the effect of lesion severity on cognitive impairment. We also found that age significantly predicted performance on executive tests and the majority of our other cognitive measures, except vSTM and GNT. Age was the only predictor for fluid intelligence. This latter finding suggests that age plays a role in executive performance over and above the contribution of CR proxies in patients with focal frontal lesions. Overall, our results suggest that the CR proxies do not appear to modify the relationship between cognitive impairment and frontal lesions.

## Highlights

• Effect of cognitive reserve (CR) proxies in focal unilateral lesions.
• NART IQ accounts for a larger proportion of variance in executive and naming skills.
• Age predicts performance on executive, fluid intelligence, speed and perception tests.
• Education and NART IQ did not modify the effect of lesion severity on cognitive impairment.

## 1 Introduction

It is well known that the cognitive response to brain damage caused by stroke, tumour, trauma, dementia and/or age-related changes can vary across individuals to a considerable degree (e.g., Stern, 2009; Lindenberger et al., 2013). The Cognitive Reserve (CR) hypothesis was put forward to account for some of the reported variability in cognitive performance and suggests that the effects of age-related changes or brain damage can be mitigated by the premorbid efficiency, capacity and flexibility of cognitive processing (e.g., Stern, 2002; Jones et al., 2011; Barulli and Stern, 2013; Levi et al., 2013). It has also been proposed that the effectiveness of cognitive processing can be shaped by life experiences (Stern, 2012). Several proxies have been used to estimate CR. These are thought to ‘protect’ against the impact of brain damage and include: education, socio-economic status, occupational achievement and engagement in cognitively and socially stimulating activities (Suchy et al., 2011; Levi et al., 2013; Liu et al., 2013; Okonkwo et al., 2014; for a review see Arenaza-Urquijo et al., 2015).

The most commonly used proxy of CR is education (e.g., Bennett et al., 2003; Farfel et al., 2013; Sumowski et al., 2013). Education encompasses the accumulated knowledge and skills gained through formal schooling. It is easy to quantify and has been shown to be a good predictor of healthier lifestyle and economic prosperity (e.g., Mirowsky and Ross, 2005). In patients with similar levels of pathology, high education has been associated with less cognitive impairment than in patients with low education (e.g., Bennett et al., 2003; Ngandu et al., 2007; Roe et al., 2007). Another proxy of CR is literacy attainment, often assessed using the National Adult Reading Test (NART; Nelson and Willison, 1991) or other single word reading tests, as they are assumed to reflect premorbid intelligence (Wiens et al., 1993). Higher NART IQ has been associated with greater CR capacity (Tucker and Stern, 2011, Stern, 2012).

The concept of CR has been investigated primarily in neurodegenerative disorders, traumatic brain injury and healthy aging. Several studies have reported that individuals with similar brain pathology demonstrate differences in their cognitive impairment, depending on whether they have higher or lower levels of education and/or NART IQ (e.g., Grafman et al., 1986; Bennett et al., 2003; Stern, 2006; Singh‐Manoux et al., 2011; Serra et al., 2014; Bozzali et al., 2015). For example, low education increases the risk of dementia whilst high education and NART IQ protects against dementia, particularly the onset of Alzheimer's disease (AD, Schmand et al., 1997; Meng and D’Arcy, 2012; Lo, and Jagust, 2013; see for a review Xu et al., 2015). High education has been shown to attenuate the decline in attention, speed and memory performance in patients with multiple sclerosis (Sumowski et al., 2014). Raymont et al. (2008) reported that higher pre-morbid intelligence, assessed using the armed forces qualification test, was the strongest deterrent against cognitive decline in patients with penetrating head injury.

However, few studies have investigated the effects of CR on cognitive performance in aetiologies such as stroke (for a review see Nunnari et al., 2014) or brain tumour where the lesions are focal compared to the diffuse lesions associated with slow progressive diseases such as Alzheimer's disease. In stroke, it has been reported that education protects against global cognitive decline (e.g., Sachdev et al., 2004; Elkins et al., 2006; Zieren et al., 2013) or severity of aphasia (González-Fernández et al., 2011). In brain tumour, although data have not been reported to suggest that education can attenuate cognitive impairment, age and tumour location (frontal) have been shown to predict cognitive outcome on speed, executive and working memory tasks (Kaleita et al., 2004).

Relatively little is known about whether CR may differentially affect performance on different cognitive measures. There is some preliminary evidence suggesting that certain cognitive abilities may be more susceptible than others to the mitigating effects of CR. *High education* has been associated with better performance in stroke patients on tests of language, perception and memory, but not executive functioning, once white matter integrity had been taken into account (Ojala-Oksala et al., 2012). Higher rates of decline in AD patients with *lower education* have been reported on memory and executive tasks but not abstract reasoning, visual-spatial skills or language (Scarmeas et al., 2006).

Importantly, the relationship between CR and different cognitive measures may also be dependent on the proxy used to estimate CR. In a large cohort study including healthy older adults and patients with possible dementia, Jefferson et al. (2011) found that education was primarily related to performance on global cognition, episodic and semantic memory, and perception. In contrast, NART IQ was found most strongly associated with working memory but also global cognition and episodic memory. Siedlecki et al. (2009) found no significant correlation between education and NART IQ in healthy controls and suggested that these two proxies may account for different elements of the variance in cognitive performance. Using path analysis on data from the MRC National Survey of Health and Development cohort, Richards and Sacker (2003) demonstrated three independent paths from childhood cognition, and educational and occupational attainment to CR using the NART as an index. Furthermore, when examining the relationships between brain volume and CR proxies in healthy adults over a two-year period, Persson et al. (2016) reported that larger baseline brain volumes predict greater increases in fluid intelligence. In contrast, no relationships between literacy attainment and brain volumes were found. Researchers have argued that more than one CR proxy measure should be considered, as CR is the result of a combination of life experiences and activities (e.g., Stern, 2009; Tucker and Stern, 2011). One CR proxy is unlikely to provide an absolute measure of CR. A recent meta-analysis examining the influence of education, occupational attainment, and involvement in cognitively stimulating activities (e.g., crosswords, playing bridge) in healthy individuals demonstrated that while different CR proxies are associated with one another, they also offer a unique contribution to CR (Opdebeeck et al., 2016).

The effects of CR may also be dependent on the specific brain regions that are damaged. For example, Robertson (2014) suggested that the structural and functional integrity of the lateral surface of the right prefrontal cortex and/or the right inferior parietal cortex may play a crucial role in CR. Higher levels of CR have also been associated with the concept of scaffolding, a lifelong process that involves the use and development of complementary, alternative neural circuits to achieve a particular cognitive goal (Alexander et al., 1997, Perneczky et al., 2006). The concept of scaffolding has been developed in the theoretical context of the Scaffolding Theory of Aging and Cognition (STAC) suggesting that, in healthy aging and in pathological brain damage, scaffolding can compensate for cognitive decline (Reuter-Lorenz and Park, 2014). Importantly, Park and Reuter-Lorenz (2009) proposed that scaffolding processes largely reside in the prefrontal cortex. Thus, damage to the prefrontal cortex may have a detrimental effect on the compensation provided by CR.

Surprisingly, the studies conducted so far have not investigated whether there are specific brain areas critical for CR. The non-specific nature of brain-related changes in neurodegenerative diseases, traumatic brain injury and healthy aging limits our ability to draw conclusions as to which specific brain areas may contribute to CR. Examination of patients with more focal brain lesions, such as those resulting from stroke and tumour, may overcome such limitations. However, the stroke and tumour studies conducted so far have included patients with lesions not restricted to specific cortical areas. Thus, the high degree of variability in the patients’ cognitive performance inevitably reduces one's ability to draw conclusions regarding the interaction between CR and brain lesion.

To our knowledge, our study is the first to investigate the independent effects of education and NART IQ on measures of executive function, fluid intelligence, speed of information processing, verbal short term memory (vSTM), naming and perception in a large sample of patients with focal, unilateral frontal lesions. Our study also investigated for the first time whether CR might safeguard against focal neuropathology and moderate cognitive impairment across these various cognitive measures.

## 2 Material and methods

### 2.1 Participants

Data from 164 patients who had attended the Neuropsychology Department of the National Hospital for Neurology and Neurosurgery, Queen Square, London, were retrospectively screened for study eligibility. All patients had a unilateral lesion confined to the frontal region resulting from stroke or brain tumour. All tumour patients had undergone lesion resection prior to neuropsychological assessment. Our exclusion criteria were: i) age at the time of cognitive testing ≥80 years due to the availability of age-matched healthy control data and standardised age norms; ii) current or previous psychiatric disorders; iii) previous neurological disorders including strokes or tumours; iv) presence of metastatic tumours; v) previous chemotherapy; vi) gross visual (i.e., cortical blindness), perceptual (i.e., neglect; agnosia), motor (i.e., hemiplegia) or language (i.e., dysphasia) impairment; vii) previous head trauma; viii) history of alcohol or drug abuse; ix) no MRI or CT scan results available; x) no or limited neuropsychological data available; xi) a score <5th percentile on a test of general intelligence (WAIS-III, Wechsler, 1997; WAIS-R, Wechsler, 1981; or Raven's Advanced Progressive Matrices, RAPM, Raven, 1976); xi) a score of <2 on a measure of frontal lesion severity (see below, ‘Frontal lesion severity’ section). Non-native English speakers were only included in the study if they obtained a score ≥25th%ile on the National Adult Reading Test (NART IQ, Nelson, 1982). This was to ensure that their English abilities were sufficient to cope with task demands.

Application of our exclusion criteria resulted in the data of 86 frontal patients being included in the study (stroke, n=22; high grade tumours, n=18; low grade tumours; n=22; meningioma n=24). Some clinical and cognitive aspects of these patients have been previously reported (MacPherson et al., 2010, MacPherson et al., 2016, Robinson et al., 2012, Robinson et al., 2015, Murphy et al., 2013, Cipolotti et al., 2015a).

Data from a group of 142 healthy controls who did not significantly differ in terms of age, gender, NART IQ and years of full-time education to the frontal group were also reviewed (see below). The study was approved by the National Hospital for Neurology and Neurosurgery and the Institute of Neurology Joint Research Ethics Committee (UK).

### 2.2 Neuroimaging investigation

MRI data were available for 76 of the frontal patients and CT data for the remaining 10. Hard copies or digital records of all scans were reviewed by two independent neurologists (MB and BS) who were blind to the experimental results. Digital brain MRI scans were obtained on systems operated at 0.5, 1.5 or 3 T and included the acquisition of an axial dual-echo and an axial and coronal T1-weighted scan. CT scans were obtained using spiral CT systems. Axial images were collected with an effective slice thickness of 5 mm and pitch of 1.5. Only T1-weighted MRI scans (or CT scans when MRI was not available) were used for the assessment of frontal lesions. We conducted an analysis of the total frontal lesion volumes only for a subset of patients (N=43). These were the patients for whom we obtained the largest number of MRI scans at the same magnetic strength (1.5T).

### 2.3 Frontal lesion severity

**Fig. 1:**
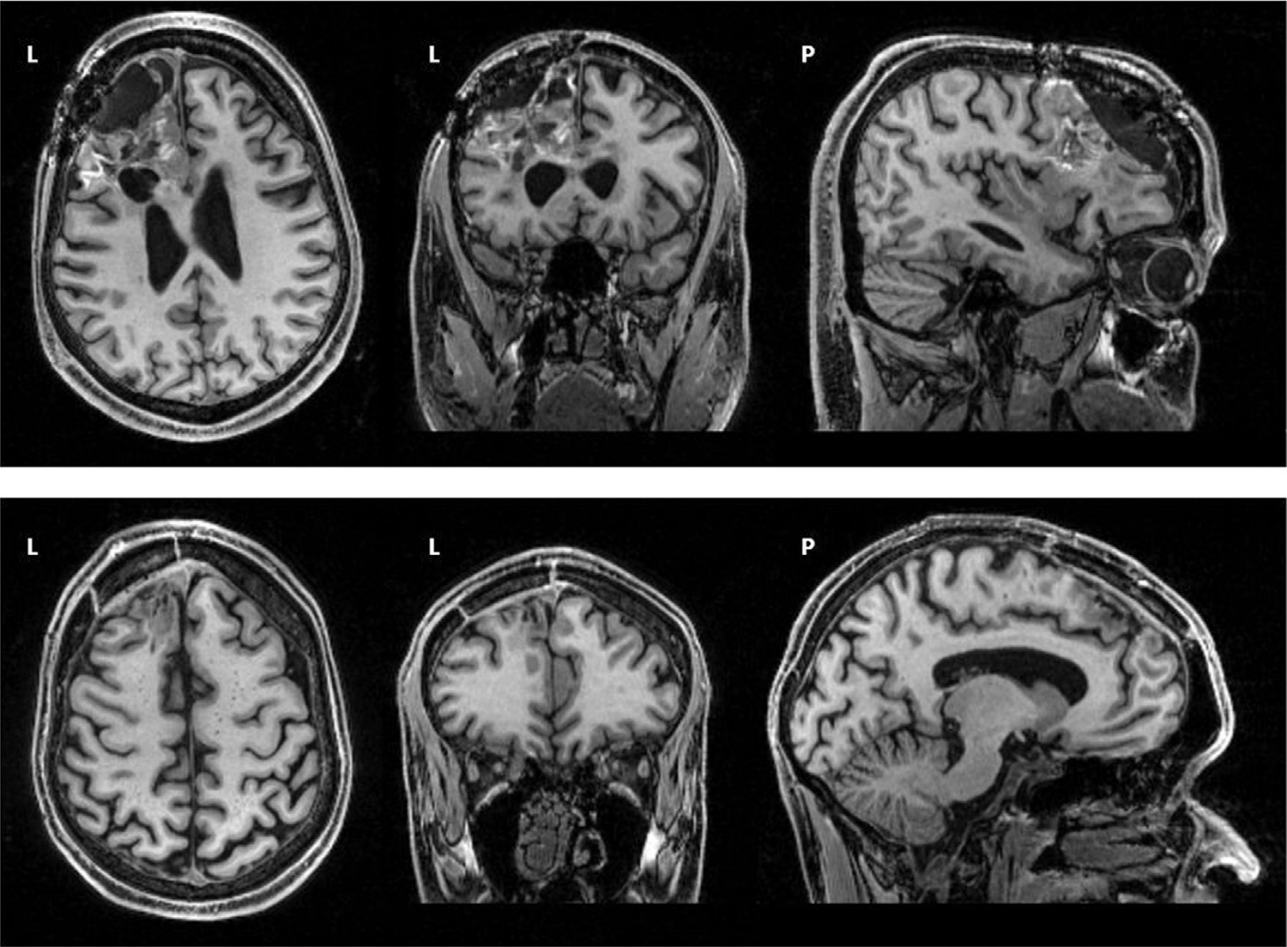
Examples of different frontal lesion severity. Top panel shows T1-weighted MR images from a patient with a severe/very severe frontal damage (total score=38). Bottom panel shows images from a patient with minimal/moderate damage (total score=6).

The lesion localization method adopted has been described in detail in our previous work (e.g., MacPherson et al., 2010; Murphy et al., 2013; Cipolotti et al., 2015a, Cipolotti et al., 2015b; Robinson et al., 2015). The exclusion criteria and lesion assessment guidelines were based on detailed anatomical localization using standard atlases (Duvernoy et al., 1991). Briefly, each frontal patient was coded for the presence of lesion and oedema in nine left and nine right frontal subregions (18 subregions in total). A subregion was coded as damaged if at least 25% was affected. Lesions were distributed throughout the prefrontal cortex. A measure of the severity of frontal lesions was obtained for each patient, using T1-MRI or CT scans. Lesion severity was assessed by visual rating (MB and BS) of each of the 18 frontal subregions based on a scale whose scores ranged from 0 to 4 (0=absence of lesion; 1=minimal damage; 2=moderate damage; 3=severe damage; 4=very severe damage). We summed the severity scores for each of the 18 frontal subregions to produce a total frontal lesion severity score (out of a possible 72); the higher the score indicated the greater the frontal lesion severity (see Fig. 1 for examples).

### 2.4 Volumetric analysis

A volumetric lesion analysis was also conducted on a subsample of 43 frontal patients who had a digital MRI scan (stroke=11, low grade tumours=12, high grade tumours=9 and meningioma=11) using a semi-automated local thresholding contouring technique (Jim 5.0, Xinapse System, Leicester, UK, http://www.xinapse.com/). The frontal lesion volume was then calculated for each patient.

### 2.5 Cognitive investigation

All frontal patients and healthy controls had previously taken part in a single neuropsychological assessment and their cognitive performance on well-known clinical tests with published standardised normative data was retrospectively considered. While a single test was administered to assess fluid intelligence, speed of information processing, naming, verbal short-term memory (vSTM), and visual perception (see Table 1 for the tests administered), the data from two tests assessing executive abilities were available (Phonemic fluency ‘S’, Tombaugh et al., 1999 and the Stroop Colour-Word Test, Trenerry et al., 1989). Due to the retrospective nature of our study, certain data were not available for some participants. The number of frontal patients who had data for each cognitive measure were as follows: executive function: Phonemic fluency: N=80; and Stroop Colour-Word Test: N=53; fluid intelligence: N=55; speed of information processing: N=55; naming: N=83; vSTM: N=36; and visual perception: N=69. The number of healthy controls who had data for each cognitive measure were as follows: executive function: phonemic fluency: N =45; and Stroop Test: N=60; fluid intelligence: N=82; speed of information processing: N=86; naming: N=139; vSTM: N=16; and visual perception: N=25. A pairwise deletion method was used with no substitutions made to the dependent variables.

### 2.6 Cognitive reserve proxies

Education and literacy attainment were adopted as our two proxies of CR, with these data available for all frontal patients and healthy controls. We operationally defined educational attainment as the number of full-time years in formal education. In keeping with previous studies, a proxy of literacy attainment was obtained using a test of single word reading (e.g., Scarmeas et al., 2006; Stern et al., 2008). This was based on the corresponding IQ score on the NART.

### 2.7 Statistical analysis

Unless stated otherwise, the statistical analyses were carried out using IBM SPSS Statistics 21 (http://www01.ibm.com/software/analytics/spss/). A Pearson's product moment correlational analysis was conducted to examine whether there was a significant relationship between our frontal lesion severity score based on visual ratings and our volumetric lesion analysis on a subsample of 43 frontal patients. Independent samples *t*-tests investigated whether the frontal patients and healthy controls, significantly differed in terms of age, years of education and NART IQ. Levene's tests were used to assure equality of variances. NART IQ and time between damage and assessment violated this assumption. Hence we used the results which did not assume equality of variances. Analyses of covariance (ANCOVAs) with age, years of education and NART IQ as covariates were conducted to investigate whether performance on our cognitive measures significantly differed between the frontal group and healthy controls. To examine the effect of lesion severity on cognitive performance, frontal patients were subdivided into high and low lesion severity groups and their performance was compared using independent samples *t*-tests.

To investigate the effect of our two CR proxies on performance on the *cognitive measures* in our frontal patients*,* we fitted multiple linear regression models for each of the cognitive measures using R function ‘lm’. In the first three steps of the analysis, chronicity (step 1), age (step 2) and severity of lesion (step 3) were each separately entered as continuous covariates.

In the fourth step, either education (step 4a) or NART IQ (step 4b) was entered as a predictor variable. This fourth step allowed us to examine the contributions of our CR proxies, education and NART IQ, to cognitive performance, over and above any effect of chronicity, age and lesion severity. In step 5, education and NART IQ were entered into the same model to examine whether they independently contributed to cognitive performance. Lastly, in step 6, either the interaction term between lesion severity and education (step 6a) or lesion severity and NART IQ (step 6b) was added. This was added to determine whether any association between lesion severity and cognitive performance differed depending on whether education or NART IQ was high or low.

For all models, the contribution and significance of each predictor was estimated at each step. Throughout the results section, we report standardized beta values. The p-value was Bonferroni corrected for multiple comparison (0.05/7=0.007) as seven cognitive measures were considered.

Missing data for our chronicity and lesion severity covariates were treated by a simple two-stage method of imputation and sensitivity analysis (Allerhand et al., 2014). In the first stage, each independent variable was regressed onto the other independent or auxiliary variables that provided coverage. The regression model for each variable was used to predict a value for each case where data were missing. The value that was imputed was then the most plausible in the sense of the most expected by the other variables. Values were not imputed for the dependent variables as imputation of these missing data is not considered to provide additional information, and may introduce additional error (von Hippel, 2007).

In the second stage, the results of the final model were assessed for sensitivity to the imputed values. A set of 100 samples were generated in which each value to be imputed was randomly drawn from a uniform distribution bounded by the prediction interval around the value predicted in the first stage. These data were all analyzed in the same way and the distribution of each estimated regression coefficient was compared against the confidence intervals of its original estimate.

## 3 Results

### 3.1 Neuroimaging investigation

Lesion definition was in an excellent range of inter- and intra- rater reliability (inter-class correlation=0.967; intra-class correlation=1.000). We first assessed whether scores of frontal lesion severity (based on visual ratings) were correlated with lesion volumes in the subsample of 43 patients with digital MRI data. As expected, our frontal lesion severity measure correlated highly with our volumetric frontal lesion analysis (r=0.676, *p*<0.001), indicating that a higher frontal severity score was associated with higher frontal lesion damage in our subgroup.

### 3.2 Cognitive investigation

The frontal group and healthy controls did not significantly differ in terms of age (t(226)=0.298, *p*=0.766), years of full-time education (t(226)=0.867, *p*=0.387) and NART IQ (t(136.706)=−0.678, *p*=0.499; See Table 2a). In our frontal patients, age did not significantly correlate with education (r=−0.17, *p*=0.12) or NART IQ (r=0.04, *p*=0.69). However, education and NART IQ correlated highly with one another (r=0.48, *p*<0.001).

#### 3.2.1 Effects of frontal lesions

Frontal patients significantly differed from healthy controls on all neuropsychological measures except the Stroop Test, as shown using ANCOVAs with age, years of education and NART IQ as covariates (executive function: fluency *F*(1,120)=13.158, *p*<0.0001; and Stroop *F*(1,108)=0.708, *p*=0.410; fluid intelligence *F*(1,142)=13.408, *p*<0.001; speed of information processing *F*(1,136)=6.806, *p*=0.010; naming *F*(1,217)=9.584, *p*=0.002; vSTM *F*(1,47)=4.375, *p*=0.042 and perception (*F*(1,89)=25.637, *p*<0.001). For all significant differences, with the exception of perception, frontal patients performed significantly poorer than healthy controls. For perception, frontal patients obtained a better score than healthy controls, although scores were close to ceiling for both samples (see Table 2b).

#### 3.2.2 Effect of severity of lesion

Qualitatively, 60 of the 78 of the patients that could not be included in our retrospective study had limited or no neuropsychological data available because they were not able to cope with the demands of our cognitive battery. The largest majority of these patients had very large frontal lesions, suggesting that severity of lesion is a powerful determinant of cognitive performance. The sample of frontal patients who were included in our retrospective study had lesion severity scores ranging from 2 to 66 out of a possible 72. Noticeably, we had few patients with the most severe scores. To investigate formally the effect of lesion severity on cognitive performance, we subdivided into two groups: patients with a high lesion severity score (≥25); and patients with a low lesion severity score (≤5). The means and standard deviations for these two groups are demonstrated in Table 2a, Table 2b. We found no significant differences between these two patient groups in terms of age (*t*(43)=1.079, *p*=0.29), time between damage and assessment (*t*(12.45)=1.287, *p*=0.22), education (*t*(20.90)=−0.603, *p*=0.55) or NART IQ (*t*(43)=−1.711, *p*=0.09). Independent samples *t*-tests revealed that the frontal patients with high lesion severity performed significantly more poorly than the frontal patients with low lesion severity on fluency (*t*(38)=−2.473, *p*<0.05) and speed of information processing (*t*(25)=2.290, *p*<0.05) tasks. No significant differences were found for fluid intelligence (*t*(35)=−1.156, *p*=0.26), naming (*t*(42)=−0.154, *p*=0.61) or perception (*t*(33)=0.496, *p*=0.62). Due to the relatively small sample sizes for Stroop (high severity: N=9 and low severity: N=15) and vSTM (high severity: N=3 and low severity: N=15), no formal statistical analyses could be carried out for these cognitive measures. Importantly, the patients with high lesion severity scores obtained scores that were numerically lower.

#### 3.2.3 Predictors of cognitive functioning

For each of our linear regression models, we examined multicollinearity using the variance inflation factor (VIF). In all instances, VIF was below 2, suggesting that there were not high intercorrelations among our predictor variables. Table 3 shows the results of the multiple linear regression models testing for the effects of education and NART IQ on each cognitive test with chronicity, age and frontal lesion severity as covariates; including education only (step 4a); NART IQ only (step 4b); education and NART IQ (step 5); and adding the interaction term between lesion severity and education (step 6a) or NART IQ (step 6b). In these analyses, lesion severity was a continuous variable.

##### 3.2.3.1 Executive measures (fluency and Stroop)

For both *executive measures*, age and NART IQ independently contributed to the fit of the model. In contrast, chronicity or frontal lesion severity made no significant contribution to the model either as main effects or as interaction terms with NART IQ or education. In the case of fluency, the model including NART IQ explained 27% of the variance compared to 16% before NART IQ was entered. For the Stroop test, the model including NART IQ explained 54% of the variance compared to 10% before NART IQ was entered.

##### 3.2.3.2 Fluid intelligence (Ravens Advanced Progressive Matrices: RAPM)

Education (step 4a) and NART IQ (step 4b) contributed to the fit of the model. However, when both education and NART IQ were entered into the same model (step 6), neither cognitive proxy continued to predict performance, leaving age as the only significant predictor. The final model explained 36% of the variance with 12% of the variance explained by age. Chronicity and frontal lesion severity did not significantly contribute to any of the models as main effects or interacting with education or NART IQ.

##### 3.2.3.3 Speed of processing (Trail Making Part A: TMT-A)

Only age significantly contributed to the fit of the model explaining 40% of the variance on TMT-A. In contrast, chronicity, frontal lesion severity, education and NART IQ made no significant contributions to the model at any stage.

##### 3.2.3.4 Verbal short-term memory (digit span: vSTM)

None of our covariates (chronicity, age and frontal lesion severity) or predictors (education or NART IQ) made significant contributions to performance on the digit span task.

##### 3.2.3.5 Naming (Graded Naming Test: GNT)

Both education and NART IQ significantly contributed to performance on the GNT. However, their contributions were not independent of one another, as entering both education and NART IQ into the same model removed the association between education and GNT performance, leaving only NART IQ as a significant predictor. Chronicity, age and lesion severity did not contribute to the model as main effects or interaction terms with education or NART IQ. NART IQ accounted for 48% of the variance on the GNT.

##### 3.2.3.6 Visual perception (Object Decision test from the Visual Object and Space Perception Battery: OD)

Only age significantly predicted performance on the Object Decision test, accounting for 9% of the variance, with no significant contributions to the model from chronicity, frontal lesion severity, education or NART IQ.

**Fig. 2:**
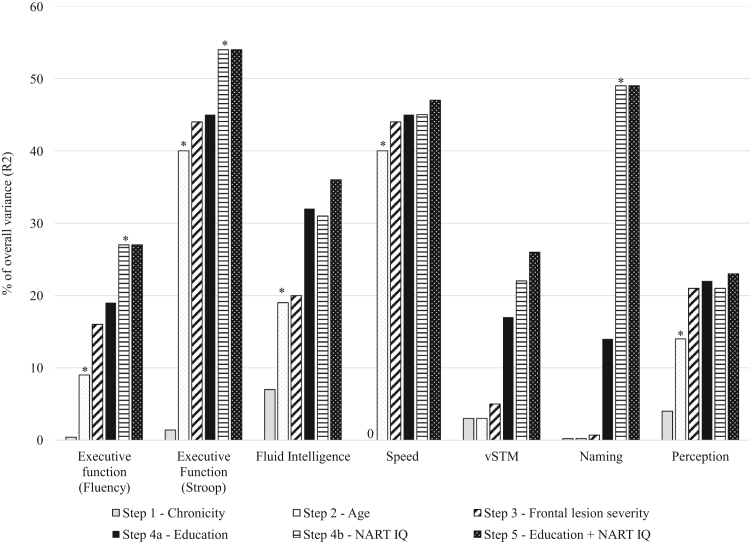
Percentage of total variance accounted for by each step of the analysis (R^2^). The R^2^ values show the amount of variance in the cognitive measures predicted by the regression models, with the significant predictors for the final models indicated. For example, the highest increase in explained variance in the final full model for fluency performance was produced by age and NART IQ. vSTM=verbal short-term memory.

In Fig. 2, we report the percentage of the total variance R^2^ accounted for by each of the five steps entering chronicity, age, frontal lesion severity, education, NART IQ and finally education and NART IQ in the regression models for all cognitive measures. All the significant associations were in the expected direction with better cognitive performance associated with younger age and higher education and NART IQ.

Our sensitivity analyses were conducted on our final full models. They showed that none of the estimates using randomly imputed values were significantly different from the estimate obtained using the most plausible value.

## 4 Discussion

We retrospectively investigated the effects of years of education and literacy attainment based on NART IQ on the cognitive performance of a large sample of patients with focal unilateral frontal lesions. Our unilateral frontal patients were significantly impaired on measures of executive function, fluid intelligence, speed of information processing, vSTM and naming compared to healthy controls. Our investigation of the contributions of education and NART IQ, after adjusting for chronicity, age and frontal lesion severity, revealed that the two variables do not represent the same proxy measures of CR. When both education and NART IQ were entered into the same model, we found that NART predicted executive and naming performance. Neither education nor NART IQ predicted performance on fluid intelligence, processing speed, vSTM or perceptual abilities. These findings support the suggestion that CR is a multidimensional construct with different proxies offering distinct contributions to performance on different cognitive measures (Siedlecki et al., 2009, Opdebeeck et al., 2016). However, it remains inconclusive which specific cognitive domains are related to specific CR. For example, studies have reported that education and NART IQ are strongly associated with fluid intelligence (e.g., Ritchie et al., 2013; Deary and Brett, 2015) and naming (Vemuri et al., 2011) when considered separately. Robertson (2014) suggested that CR predicts performance on frontal executive tasks. However, literacy attainment has been shown to predict performance on speed of information processing in patients with multiple sclerosis (Benedict et al., 2010) and a latent executive variable in traumatic brain injury (Green et al., 2008). Educational attainment in healthy aging has been found to predict performance on executive abilities and working memory (e.g., Meguro et al., 2001) and memory and perception (Jefferson et al., 2011). Education has been shown to predict performance on executive tasks and memory in AD (Scarmeas et al., 2006) but not in stroke (Ojala-Oksala et al., 2012). Moreover, our retrospective study has the disadvantage of having relatively smaller sample sizes for certain cognitive measures (e.g., vSTM N=36) compared to other measures. Therefore, caution should be taken when concluding that NART and education are selectively unrelated to vSTM. Future prospective work is needed to examine further the relationship between CR proxies and cognition.

We found that age significantly predicted performance on executive tests and on the majority of our other cognitive measures, exceptions being vSTM and GNT. For fluid intelligence, when both education and NART were entered together in the model, only age remained a significant predictor. We have previously reported that age and NART IQ significantly predicted performance on a variety of cognitive measures in patients with frontal lesions, some of whom have participated in the current study (Cipolotti et al., 2015a). Previous research has reported significant age-related decline in fluid intelligence performance in healthy adults (Raz et al., 2007, Elderkin-Thompson et al., 2008). We have also previously documented that age in frontal patients significantly predicts the magnitude of the impairment in fluid intelligence (RAPM, Cipolotti et al., 2015b). Our current findings further confirm the important role of age in executive performance in the context of frontal lesions over and above the contribution of CR proxies.

Interestingly, in our current study, our regression analyses suggest that the severity of our patients’ frontal lesions does not appear to play a critical role on their executive and fluid intelligence performance. However, more broadly, frontal lesion severity appears to contribute to cognitive performance. Qualitatively, we noted that the large number of patients who were unable to cope with the demands of our cognitive battery, and hence were not included in our retrospective study, tended to have rather large frontal lesions. Moreover, when we compared the patients with high and low lesion severity, we found that patients with high lesion severity performed significantly more poorly on fluency and speed of processing tasks than patients with low lesion severity. They also obtained poorer scores on the other cognitive tests, the only exception been perception. Importantly, the two groups of patients were matched on education and NART IQ. Future prospective studies are necessary to examine the role of lesion severity on cognition.

Our results also revealed that the effects of education and/or NART IQ on our cognitive measures did not interact with lesion severity. Thus, we found no evidence that the effect of lesion severity on cognitive impairment is altered by either CR proxy in our frontal patients. It seems unlikely that these lack of interactions simply reflect the fact that lesion severity is irrelevant and they may reflect non-linear effects when lesion severity is considered as a continuous variable. As reported above when considered a dichotomous variable, lesion severity may play a role in the cognitive performance of frontal patients. While this was not captured by our regression analyses, we had fewer patients with the most severe scores. Stern (2009) suggested that higher CR individuals are able to tolerate a greater degree of pathology for a longer time before cognitive decline presents behaviourally. However, there is also a growing body of longitudinal evidence suggesting that CR does not alter the slope of cognitive decline (Tucker-Drob et al., 2009; Singh‐Manoux et al., 2011; Zahodne et al., 2011). Our cross-sectional study has the disadvantage of not allowing us to examine the effects of our CR proxies on the rate and magnitude of cognitive decline over time. However, our results suggest that the CR proxies do not modify the relationship between cognitive impairment and frontal lesions. It might be argued that these inconsistent results are due to the type of CR proxy measures used. The two measures we used, NART IQ and education, have been commonly adopted in the literature (Valenzuela and Sachdev, 2006a, Valenzuela and Sachdev, 2006b). However, education has been criticised as a CR proxy given that the quality, availability and subjects taught varies across different countries and social groups (e.g., Jones et al., 2011). Some authors have suggested that literacy attainment may be a better marker of educational attainment (e.g., Manly et al., 2003). However, dyslexia and other learning difficulties are detrimental to literacy attainment and result in an inaccurate estimate of CR. Hence, some authors suggest that education is the better proxy (e.g., Ikanga et al., 2016).

Alternatively, while CR allows individuals to cope more successfully with healthy and pathological age-related changes in the brain (Stern, 2009), it may not have the same neuroprotective benefit in the context of focal brain damage due to brain tumour or stroke, as in the case of our patients. Perhaps a critical difference is that there may be more plasticity and functional reorganization in healthy aging and in the neurodegenerative conditions due to their slow progressive nature (Morris, 2005, Ryan and Rossor, 2011). It may be that the effect of the CR proxies can facilitate functional reorganization to compensate for brain damage, but only when the disease process is slow (Stern, 2006). Instead, stroke and tumour are usually associated with a much more rapid disease process, hence the limited effect of the CR proxies.

It is also possible that our patients’ frontal damage hindered the degree of compensation provided by our two proxies. This notion is broadly in keeping with the view that the prefrontal cortex compensates for declines in cognitive functioning (Park and Reuter-Lorenz, 2009) and that the right prefrontal cortex, in association with other areas, mediate some the CR effects (Robertson, 2014). Unfortunately, we did not have the opportunity to investigate the effects of our two CR proxies in patients with non-frontal lesions. This would have allowed us to directly compare whether the degree of variance accounted for by education and NART IQ is reduced in frontal patients when compared to non-frontal patients, as suggested by the STAC model.

We acknowledge a number of limitations of our retrospective study. We were unable to examine the influence of CR on memory abilities due to insufficient data. We could not assess our patients’ lifestyle factors which may also mitigate the occurrence of cognitive decline and differentially modify individuals’ performance on different cognitive measures. Additionally, given the heterogeneous neuroimaging data available, we could not investigate parameters such as white matter intensities and cortical atrophy, known to associate with CR and cognitive performance (e.g., Brickman et al., 2011; Murray et al., 2011). However, a major strength of our study is that, to our knowledge, it is the first study to systematically investigate the effects of two CR proxies on the performance of patients with focal unilateral frontal lesions across several cognitive measures.

Overall, our data suggest that in the context of prefrontal lesions, the protective effects of education and NART IQ are limited to specific cognitive measures. Unlike previous studies reporting that CR can compensate for brain pathology due to healthy aging or neurodegenerative diseases, our data suggest that CR does not show the same ‘protection’ against the impact of focal brain damage involving the frontal lobes. Future work is needed to examine further the complex relationship between CR, age and the presence of a frontal lesion when attempting to understand the resulting impairments in executive tasks.

## Figures and Tables

**Table 1:** Cognitive tests corresponding to each cognitive measure.

**Cognitive measures**	**Tests**	**Scores used**	**References**
Executive Function	Phonemic fluency 'S' (Fluency)	Number of words produced	Tombaugh et al., 1999
	The Stroop Colour-Word Test (Stroop)	Number of colour-words correctly named in 2 min. If participants correctly named all colour-words in less than 2 min, their score was prorated to indicate the number they would have achieved in 2 min.	Trenerry et al., 1989; Cipolotti et al., 2016
Fluid Intelligence	Raven's Advanced Progressive Matrices (RAPM)	Number of correct responses (out of 12)	Raven et al., 1998
Speed	Trail Making Test Part A - time (TMT-A)	Number of seconds to complete	Reitan, 1992
vSTM	Digit span subtest from the WAIS-III	Number of sequences correctly repeated (out of 30)	Wechsler, 1997
Naming	Graded Naming Test (GNT)	Number of pictures correctly named (out of 30)	McKenna and Warrington, 1983
Perception	Object Decision Test from the Visual Object and Space Perception Battery (VOSP)	Number of silhouettes correctly identified (out of 20)	Warrington and James, 1991

Legend: vSTM=verbal short-term memory.

**Table 2a:** Demographic and descriptive results for CR proxies.

	HC (N=142)		Frontal group (N=86)					
			All		High lesion severity		Low lesion severity	
	***M***	***SD***	***M***	***SD***	***M***	***SD***	***M***	***SD***
**Age**	46.18	15.66	46.80	14.49	52.73	13.58	47.37	16.66
**Time between damage and assessment (months)**	N/A	N/A	13.94	28.29	31.94	57.36	11.27	11.69
**Years of education**	13.57	2.77	13.91	2.96	13.27	3.65	13.90	2.52
**NART IQ**	109.12	8.40	108.13	11.88	103.27	11.60	11.76	2.15

Note: HC=Healthy controls; M=Mean; SD=Standard deviation; N/A=Not applicable; NART=National Adult Reading Test.

**Table 2b:** Descriptive results for the cognitive measures.

	HC (N=142)			Frontal group (N=86)					
				All		High Lesion Severity		Low Lesion Severity	
	***M***		***SD***	***M***	***SD***	***M***	***SD***	***M***	***SD***
**Executive function – fluency**	16.93		4.92	13.35	6.14	11.83	6.20	15.78	5.61
**Executive function - Stroop**	98.57		29.68	96.43	34.31	82.19	30.68	107.04	39.11
**Fluid intelligence**	8.87		2.10	7.57	2.47	6.84	2.85	8.24	1.92
**Speed**	32.08		11.15	38.61	16.71	45.02	21.90	32.78	8.27
**vSTM**	20.69		4.57	17.67	4.09	14.86	2.73	18.50	3.37
**Naming**	21.78		4.08	19.95	4.82	19.17	5.00	20.13	4.96
**Perception**	18.44		1.08	19.45	0.76	19.70	0.57	19.65	0.61

Note: HC=Healthy controls; M=Mean; SD=Standard deviation; N/A=Not applicable.

**Table 3:** Regression models of cognitive reserve proxies on neuropsychological performance.

**Test**	**Variable**	**Step 4a**			**Step 4b**			**Step 5**			**Step 6a**			**Step 6b**		
		**(Education)**			**(NART IQ)**			**(Education+NART IQ)**			**(Severity×Education)**			**(Severity×NART IQ)**		
		***β***	**SE**	***p***	***β***	**SE**	***p***	***β***	**SE**	***p***	***β***	**SE**	***p***	***β***	**SE**	***p***
Fluency	Chronicity	0.19	0.11	0.09	0.17	0.10	0.10	0.17	0.10	0.10	0.17	0.11	0.12	0.17	0.10	0.10
(N=80)	Age	−0.28	0.11	0.01	**−0.32**	**0.10**	**0.002**	**−0.33**	**0.11**	**0.002**	−0.28	0.11	0.01	**−0.33**	**0.10**	**0.002**
	Severity	−0.25	0.11	0.02	−0.19	0.10	0.07	−0.19	0.10	0.07	−0.21	0.11	0.06	−0.21	0.10	0.05
	Education	0.16	0.11	0.13				−0.10	0.12	0.93	0.21	0.11	0.06			
	NART				**0.34**	**0.10**	**0.001**	**0.34**	**0.12**	**0.005**				**0.36**	**0.10**	**0.001**
	Severity×Education										−0.18	0.11	0.10			
	Severity×NART													−0.09	0.09	0.31
Stroop	Chronicity	0.19	0.11	0.09	0.16	0.10	0.18	0.16	0.10	0.12	0.19	0.11	0.09	0.15	0.10	0.16
(N=53)	Age	**−0.61**	**0.11**	**<0.0001**	**−0.67**	**0.10**	**<0.0001**	**−0.68**	**0.10**	**<0.0001**	**−0.61**	**0.11**	**<0.0001**	**−0.66**	**0.10**	**<0.0001**
	Severity	−0.18	0.11	0.10	−0.17	0.10	0.10	−0.17	0.10	0.10	−0.17	0.12	0.17	−0.18	0.10	0.08
	Education	0.12	1.11	0.26				−0.53	0.12	0.65	0.13	0.11	0.27			
	NART				**0.33**	**0.10**	**0.002**	**0.36**	**0.12**	**0.003**				**0.34**	**0.10**	**0.002**
	Severity×Education										−0.02	0.13	0.89			
	Severity×NART													0.08	0.14	0.55
RAPM	Chronicity	0.16	0.11	0.16	0.19	0.11	0.08	1.51	0.11	0.17	0.15	0.11	0.18	0.19	0.11	0.09
(N=55)	Age	−0.28	0.11	0.01	**−0.36**	**0.11**	**0.001**	**−0.31**	**0.11**	**0.006**	−0.28	0.11	0.01	**−0.36**	**0.11**	**0.001**
	Severity	−0.92	0.11	0.39	−0.02	0.11	0.82	−0.05	0.11	0.64	−0.10	0.11	0.35	−0.03	0.11	0.79
	Education	**0.37**	**0.11**	**0.001**				0.26	0.13	0.04	**0.36**	**0.12**	**0.003**			
	NART				**0.35**	**0.11**	**0.003**	0.22	0.12	0.08				**0.36**	**0.11**	**0.002**
	Severity×Education										0.05	0.12	0.67			
	Severity×NART													−0.04	0.10	0.69
Trails	Chronicity	−0.23	0.11	0.06	−0.22	0.11	0.06	−0.22	0.11	0.05	−0.21	0.11	0.07	−0.20	0.11	0.08
A	Age	**0.67**	**0.11**	**<0.0001**	**0.66**	**0.11**	**<0.0001**	**0.69**	**0.11**	**<0.0001**	**0.67**	**0.11**	**<0.0001**	**0.68**	**0.11**	**<0.0001**
(N=55)	Severity	0.22	0.11	0.06	0.21	0.11	0.06	0.21	0.11	0.06	0.18	0.12	0.12	0.21	0.11	0.05
	Education	0.05	0.11	0.34				0.13	0.12	0.30	0.03	0.11	0.80			
	NART				−0.10	0.11	0.34	−0.16	0.12	0.18				−0.13	0.10	0.23
	Severity×Education										0.11	0.12	0.37			
	Severity×NART													0.18	0.10	0.07
Digit	Chronicity	−0.10	0.22	0.66	−0.05	0.21	0.83	−0.07	0.21	0.73	−0.10	0.22	0.67	−0.05	0.22	0.83
Span	Age	−0.09	0.19	0.64	−0.21	0.19	0.29	−0.21	0.19	0.28	−0.09	0.19	0.63	−0.23	0.20	0.26
(N=36)	Severity	0.02	0.22	0.93	−0.35	0.20	0.86	0.04	0.21	0.84	0.01	0.23	0.96	−0.01	0.21	0.97
	Education	0.40	0.18	0.04				0.23	0.20	0.26	0.41	0.19	0.04			
	NART				0.48	0.18	0.01	0.37	0.20	0.07				0.51	0.19	0.01
	Severity×Education										−0.03	0.16	0.86			
	Severity×NART													0.09	0.19	0.62
GNT	Chronicity	0.01	0.11	0.90	−0.03	0.08	0.76	−0.03	0.09	0.76	0.01	0.11	0.92	−0.03	0.08	0.75
(N=83)	Age	0.10	0.11	0.37	0.02	0.08	0.84	0.02	0.09	0.81	0.10	0.11	0.39	0.01	0.08	0.92
	Severity	−0.04	0.11	0.74	0.09	0.09	0.32	0.09	0.09	0.32	−0.03	0.11	0.80	0.07	0.08	0.42
	Education	**0.37**	**0.11**	**<0.0001**				0.02	0.10	0.83	**0.38**	**0.11**	**0.001**			
	NART				**0.71**	**0.08**	**<0.0001**	**0.70**	**0.10**	**<0.0001**				**0.73**	**0.08**	**<0.0001**
	Severity×Education										−0.03	0.11	0.76			
	Severity×NART													−0.12	0.08	0.11
OD	Chronicity	−0.22	0.11	0.06	−0.20	0.11	0.08	−0.21	0.11	0.07	−0.23	0.11	0.05	−0.21	0.12	0.08
(N=69)	Age	−0.30	0.11	0.01	**−0.32**	**0.11**	**0.006**	−0.29	0.12	0.02	**−0.32**	**0.11**	**0.007**	**−0.32**	**0.11**	**0.006**
	Severity	0.26	0.11	0.02	0.25	0.12	0.04	0.23	0.12	0.05	0.30	0.11	0.01	0.24	0.12	0.04
	Education	0.08	0.11	0.46				0.16	0.13	0.24	0.11	0.11	0.36			
	NART				−0.05	0.11	0.64	−0.14	0.13	0.31				−0.05	0.12	0.70
	Severity×Education										−0.14	0.12	0.23			
	Severity×NART													−0.04	0.11	0.73

*Note.* Standardised betas are reported. Bonferroni adjusted p-value <0.007.

RAPM=Raven's Advanced Progressive Matrices; GNT=Graded Naming test; OD=Object Decision test from the Visual Object and Space Perception Battery.

Step 4a shows the effect of education on each cognitive test score. Step 4b shows the effect of NART IQ on each cognitive test score. Step 5 shows the effect of education and NART IQ on each cognitive test score. Step 6a additionally controls for the interaction term between lesion severity and education and Step 6b additionally controls for the interaction term between lesion severity and NART IQ.
